# Development of Exhausted Memory Monocytes and Underlying Mechanisms

**DOI:** 10.3389/fimmu.2021.778830

**Published:** 2021-10-28

**Authors:** Kisha Pradhan, Ziyue Yi, Shuo Geng, Liwu Li

**Affiliations:** ^1^ Department of Biological Sciences, Virginia Tech, Blacksburg, VA, United States; ^2^ Graduate Program of Genetics, Biotechnology and Computational Biology, Virginia Tech, Blacksburg, VA, United States

**Keywords:** monocyte memory, exhaustion, pathogenic inflammation, CD38, TRAM

## Abstract

Pathogenic inflammation and immuno-suppression are cardinal features of exhausted monocytes increasingly recognized in septic patients and murine models of sepsis. However, underlying mechanisms responsible for the generation of exhausted monocytes have not been addressed. In this report, we examined the generation of exhausted primary murine monocytes through prolonged and repetitive challenges with high dose bacterial endotoxin lipopolysaccharide (LPS). We demonstrated that repetitive LPS challenges skew monocytes into the classically exhausted Ly6C^hi^ population, and deplete the homeostatic non-classical Ly6C^lo^ population, reminiscent of monocyte exhaustion in septic patients. scRNAseq analyses confirmed the expansion of Ly6C^hi^ monocyte cluster, with elevation of pathogenic inflammatory genes previously observed in human septic patients. Furthermore, we identified CD38 as an inflammatory mediator of exhausted monocytes, associated with a drastic depletion of cellular NAD^+^; elevation of ROS; and compromise of mitochondria respiration, representative of septic monocytes. Mechanistically, we revealed that STAT1 is robustly elevated and sustained in LPS-exhausted monocytes, dependent upon the TRAM adaptor of the TLR4 pathway. TRAM deficient monocytes are largely resistant to LPS-mediated exhaustion, and retain the non-classical homeostatic features. Together, our current study addresses an important yet less-examined area of monocyte exhaustion, by providing phenotypic and mechanistic insights regarding the generation of exhausted monocytes.

## Introduction

Sepsis is a life threatening disease caused by dysregulated host inflammatory response leading to organ dysfunction (1, 2). Traditionally, a biphasic immune response with initial hyper-inflammation followed by immunosuppression depicted septic conditions, with the latter immuno-paralysis primarily contributing to long-term sepsis-related illness and infection (2–4). It is now accepted that both inflammatory and anti-inflammatory responses are simultaneously activated during sepsis, and dominance of one response over the other leads to dysfunctional immune response followed by exacerbated septic conditions (5). Leukocyte transcriptome data also reveal a simultaneous increase in expression of genes involved in systemic inflammatory and compensatory immunosuppressive response post-severe injury (6). Over 20% of patients that recover from sepsis either die within the first year after recovery from secondary infections or the survivors suffer from imbalances in immune responses due to prolonged inflammation and immune suppression (7). Chronic immune dysfunction in sepsis survivors is irreversible (8) emphasizing the urgency to thoroughly investigate sepsis-related immune dysfunction. However, cellular and molecular mechanisms that underlie the generation of pathogenic inflammation and immuno-suppression are still poorly understood.

Immune dysfunctions in monocytes of sepsis patients have been well-noticed through basic and translational studies. Despite a reduction of inflammatory cytokines, monocytes from septic patients exhibit elevated production of reactive oxygen species (ROS) as well as nitric oxide (NO), potentially contributing to pathogenic inflammation (9). Septic monocytes also have compromised CD86 and HLA-DR (MHC-II in mice) expressions while maintain elevated PD-L1 expressions, collectively correlated with immunosuppressive phenotypes (2, 10, 11). Flow cytometry based studies further identify that the classical monocytes (murine Ly6C^hi^, and human CD14^hi^CD16^lo^) may exhibit inflammatory natures with elevated ROS production (12, 13). In contrast, the non-classical monocytes (murine Ly6C^-^, and human CD14^lo^CD16^hi^) play important beneficial roles in maintain vasculature homeostasis and reducing pathogenic inflammation (12, 13). Infiltration of Ly6C^pos^ monocytes in early as well as late phases of sepsis has been reported in experimental murine models (5, 7). Recent studies also reveal a sharp decrease of the human non-classical monocyte populations in septic patients as well as patients with severe COVID-19 (7, 14, 15). Despite these important phenotypic observations, mechanisms responsible for the development of pathogenic inflammatory and immuno-suppressive classical monocytes are still poorly defined.

Previous *in vitro* studies with cultured monocytes recapitulating septic conditions almost solely focused on the aspect of suppressed expression of inflammatory cytokines commonly known as endotoxin tolerance (3, 16). Monocytes/macrophages with repetitive challenges of bacteria endotoxin Lipopolysaccharide (LPS) have blunted expression of inflammatory cytokines, partly due to MyD88-mediated induction of NFkB transcriptional suppressors including RelB (17, 18). However, the traditional concept of endotoxin tolerance fails to capture the cardinal features of pathogenic inflammation and immuno-suppression manifested in both human septic patients and experimental murine sepsis models. Indeed, some limited studies suggest that “tolerant” monocytes are not transcriptionally inactive. Rather, they can still robustly express “non-tolerizable” genes involved in pathogenic inflammation and immuno-suppression (6, 19, 20). Given emerging data revealing a complex “exhausted” nature of septic monocytes capable of expressing pathogenic inflammatory genes and immuno-suppressive genes, it is necessary to conduct mechanistic studies to address the generation of exhausted pathogenic monocytes, which is currently lacking in the field.

To fill this critical gap, we performed studies with integrative approaches of scRNAseq examining gene expression profiles and flow cytometry analyses of key protein markers reflecting exhausted monocytes cultured *in vitro* with repetitive challenges of higher dose LPS. We validated the expansion of exhausted Ly6^hi^ monocytes, and confirmed representative marker genes up or down-regulated in monocytes collected from septic patients in our *in vitro* culture system. Furthermore, we identified CD38 as a key marker that is robustly and persistently elevated in exhausted monocytes. CD38 is an ecto-enzyme involved in degrading and depleting both inter-cellular and intra-cellular NAD^+^, and potentially propagate inter-cellular inflammation (21–24). We also observe a drastic depletion of cellular NAD^+^, compromised mitochondria respiration and elevated cellular ROS in exhausted monocytes cultured with repetitive LPS challenges. Mechanistically, we observed that exhausted monocytes have drastically elevated STAT1 and KLF4 activation, dependent upon TRAM, the other arm of the TLR4 adaptor pathway. Together, our data reveal that sustained activation of STAT1 *via* TRAM pathway may be responsible for the generation of exhausted monocytes, with polarized induction of pathogenic inflammatory mediators such as CD38; and suppression of co-stimulatory molecule CD86.

## Materials and Methods

### *In Vitro* Cell Culture

Primary murine cells from either C57BL/6 (WT) or TRAM KO mice were used for *in vitro* cell culture. The WT mice were purchased from Jackson’s Laboratory and TRAM KO mice with C57BL/6 background were kindly provided by Dr. Holger Eltzschig (University of Texas Houston) and regularly maintained in our laboratory. All animal procedures were in accordance with the U.S. National Institutes of Health Guide for the Care and Use of Laboratory Animals and approved by Institutional Animal Care and Use Committee (IACUC) of Virginia Tech. Bone marrow cells were harvested from WT and TRAM KO mice as described previously (19, 25, 26). Primary cells were cultured in complete RPMI 1640 media with supplemented with 10% FBS, 1% penicillin-streptomycin, and 1% L-Glutamine and 10ng/mL M-CSF (PeproTech, Rocky Hill, NJ; no. 315-02). Cells were treated with either PBS (control) or high-dose LPS (100 ng/mL) for 5 days at 37°C in a humidified 5% CO2 atmosphere. Fresh complete media supplemented with M-CSF and treatments were added every 2 days as reported previously (19, 25, 26). As we previously characterized, bone marrow cells cultured under such condition were monocyte-like, loosely adherent, and did not express mature macrophage marker CD71 (27).

### Reagents

LPS (Escherichia coli 0111:B4) was purchased from Sigma-Aldrich. Primary anti-STAT1 (#9172S, 1:1000 in 5% BSA) and anti-phospho-STAT1 (#9177S, 1:1000 in 5% BSA) antibodies were purchased from Cell Signaling. Anti-β-actin (HRP) (# 47778, 1:1000 in 5% BSA) antibody was purchased from Santa Cruz. Primary anti-KLF antibody (#ab129473, 1:1000 in 5% BSA) was purchased from Abcam. N-Acetyl-L-cysteine or NAC (#A9165) was purchased from Sigma-Aldrich. Fludarabine, a STAT1 activation inhibitor (S1491) was purchased from Selleck Chemicals.

### Western Blot Analyses

Western blot was performed as previously described (25). Cells were lysed in 2% SDS lysis buffer containing protease inhibitor (Sigma, #P8340), phosphatase inhibitor 2 and 3 (Sigma, #P5726 and # P0044, respectively). Cell lysates were denatured at 95°C for 5 minutes and protein concentrations were determined using the Bio-Rad DC Protein Assay Kit (#5000112). Proteins were separated by electrophoresis using 10% Acrylamide gel and transferred to PVDF membranes. Membranes were then blocked with 5% milk for 1 h, followed by incubation with targeted primary antibodies at 4°C overnight and secondary HRP-conjugated anti-rabbit IgG antibody (#7074, 1:2000 in 5% milk) at room temperature for 1 h. Images were developed with ECL detection kit (VWR, # 490005-020), and the relative expressions of target protein were quantified with ImageJ software (NIH).

### NAD^+^ Assay

Amplite™ Fluorimetric cADPR-Ribose Assay Kit was used to determine the NAD^+^ levels following manufacturer’s instructions. Cytation^3^ Image Reader (BioTek) was used to quantify NAD+ levels.

### Flow Cytometry

On Day 5, murine monocytes were washed with PBS, harvested, and blocked in with 1:100 Fc block (BD Biosciences, #553141). Cells were then stained with fluorochrome-conjugated antibodies against Ly6C (PE-Cy7; Biolegend #128018), CD11b (allophycocyanin-Cy7; Biolegend #101226), CD86 (FITC; Biolegend # 105006), MHCII (FITC; Biolegend # 107606), PD-L1 (APC; Biolegend # 124312) and CD38 (PE; Biolegend # 102708), for 30 minutes followed by washing with FACS buffer. Finally, cells were resuspended in (Life Technologies # P3566) in FACS buffer containing Propidium Iodide (PI, 1:200). To determine intracellular ROS levels, CellROX Deep Red Reagent (Life Technologies #C1049) was added to cell cultures 45min before harvesting and the cells were also co-stained with and Live/Dead (Life Technologies # L34970). Samples were analyzed with a FACS Canto II (BD Biosciences), and data were analyzed with FlowJo (Ashland, OR).

### Seahorse Assay

Monocytes (10^6 cells/mL) were seeded in the Seahorse Bioscience XF24 (Agilent) cell culture plates and cultured for 5 days. Fresh media and treatments were added every 2 days. Cell Mito Stress Test was performed by sequentially treating the cells with 0.5 μM oligomycin, 0.6 μM carbonyl cyanide-4-(trifluoromethoxy) phenylhydrazone (FCCP) and 0.1 μM rotenone/antimycin A according to the manufacturer’s instructions.

### scRNA-seq and Data Analysis

Cell preparation and scRNAseq were performed as we previously described (27). Briefly, FACS-purified cells were resuspended in cold PBS supplemented with 0.04% BSA (VWR). About 1,000 cells were targeted for the experiment. Gel-Bead in Emulsions (GEMs) were generated using the 3′ V3 chemistry (Chromium Single Cell 3’ Reagent Kit, 10X Genomics) and RT reaction was conducted in the C1000 touch PCR instrument (BioRad). Barcoded cDNA was obtained from the GEMs by Post-GEM RT-cleanup and amplified for 12 cycles. Amplified cDNA was subjected to enzymatic fragmentation, end-repair, A tailing, adaptor ligation and 10X specific sample indexing as per manufacturer’s protocol. Libraries quality and quantity were determined using Bioanalyzer analysis. Libraries were then pooled and sequenced on Illumina HiSeq platform (Novogene). Analyses of data were performed as we previously published (28) and briefly described below. Cell Ranger (version 3.1.0) from the 10X Genomics website (https://support.10xgenomics.com/single-cell-gene-expression/software) were used to annotate mouse reference genome, perform the alignment and mapping of sequenced reads, as well as the quantification of relative levels of gene expression. The default pipeline of Seurat (version 3.1.4) in R were used to perform quality control and data normalization. Doublets as well as cells with fewer than 200 unique genes were excluded. Genes that existed in fewer than 3 cells were also removed. A small cluster of neutrophils expressing *Ly6g* was excluded. Dimensionality reduction was performed by principal component analysis (PCA), and cells were clustered through UMAP analysis as we reported (28). Differentially expressed genes with significant differences among distinct clusters were identified. Selected genes from top 200 differentially expressed genes as well as patho-physiologically relevant players involved in pathogenic inflammation and immunosuppression related to sepsis were presented on the heat-map and the dot plot. scRNAseq data sets were deposited in the Genebank with the accession number GSE182355.

### Statistical Analysis

GraphPad Prism v7.0 (GraphPad Software, La Jolla, CA) was used to generate graphs and to perform statistical analyses. Student’s *t*-test (for two groups) or one-way analysis of variance (ANOVA) (For multiple groups) was used to determine the significance, where P<0.05 was considered statistically significant.

## Results

### Generation of Exhausted Monocytes With Prolonged and Repetitive Treatments of High Dose LPS

**Figure 1:**
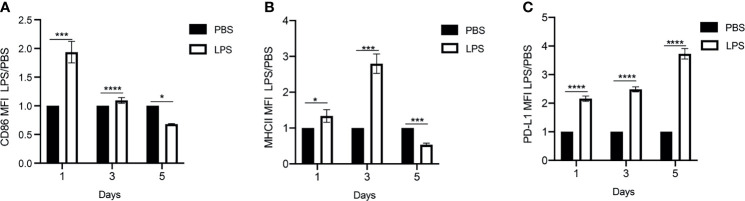
Generation of exhausted monocytes *via* repetitive LPS challenges. BMDMs were treated with PBS or high dose LPS (100ng/mL) for 5 days. **(A–C)** The Mean Fluorescent Intensity (MFI) of CD86, MHCII and PD-L1 on day 1, 3 and 5 were monitored using flow cytometry. The relative fold changes in MFIs of CD86, MHCII and PD-L1 comparing LPS-treated *vs* PBS-treated monocytes were determined and plotted in the graphs. Data were representative of three independent experiments, and error bars represent means ± SEM (n=3 for each group). *p < 0.05, ***p < 0.001, ****p < 0.0001, Student *t* test.

To monitor the exhaustion of primary monocytes to persistent challenges of bacterial endotoxin LPS, we cultured murine monocytes derived from bone marrow for 5 days in the presence of M-CSF to maintain cell survival as we described (25, 26), with the repetitive challenges with either PBS or high dose LPS (100ng/mL) on Day 0, 2 and 4. We then harvested the cultured cells at day 1, 3, and 5 for flow cytometry analyses of CD86, MHCII and PD-L1. CD86 and MHCII are key immune-enhancing mediators, while PD-L1 is a well-recognized immune suppressor. A reduction of CD86 and MHCII coupled with an elevation of PD-L1 were shown as key signatures of monocyte exhaustion closely associated with the pathogenesis of sepsis (2). As shown in **Figures 1A, B**, the initial LPS challenge led to an induction of CD86 and MHCII expression during the initial days of LPS challenge compared to PBS controls, as measured by flow cytometry analyses, consistent with previous findings (2). Expectedly, as the treatment regimen continued, the expression levels of MHCII and co-stimulatory molecule CD86 were reduced at day 5 of the treatment regimen compared to PBS controls, reminiscent of the development of LPS tolerance (**Figures 1A, B** and **Supplementary Figures S1A, B**). In sharp contrast, the expression of PD-L1 was not tolerized, and continually induced in monocytes following repetitive LPS challenge at day 3 and day 5 time points compared to PBS controls (**Figure 1C** and **Supplementary Figure S1C**). Collectively, our *in vitro* system of repetitive LPS challenges for a 5-day time period generated a representative monocyte exhaustion phenotype with reduced expression of MHCII and co-stimulatory molecule CD86, as well as continued induction of immuno-suppressor PD-L1, consistent with the exhausted monocyte phenotype observed in experimental animals and human sepsis patients (29–31). We therefore used the 5-day culture system to further characterize the exhausted monocytes in this study.

### Systems Analyses of Exhausted Monocytes Reveal Significantly Elevated CD38

**Figure 2:**
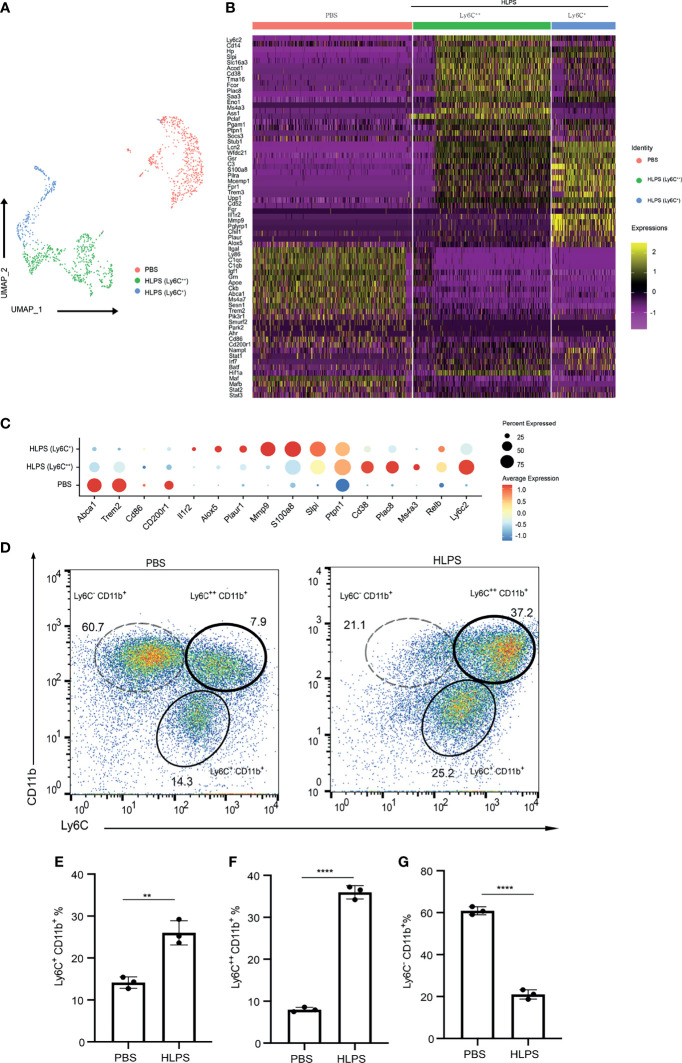
scRNAseq analyses of exhausted monocytes. **(A–C)** BMDMs were treated with PBS or high dose LPS (100 ng/mL) for 5 days and subjected to scRNA-seq analysis using Chromium Single Cell 3’ Reagent Kit V3. PBS control treated (Ly6C^-^) cluster and HLPS treated clusters (Ly6C^+^ and Ly6C^++^) are shown using UMAP plot **(A)**, Heatmap **(B)**, and Selected gene dot plot **(C)**. **(D)** Ly6C^+^ CD11b^+^, Ly6C^++^ CD11b^+^ and Ly6C^-^ CD11b^+^ populations in the cell cultures challenged with PBS or high dose LPS for 5 days were analyzed with flow cytometry. **(E–G)** The frequencies of indicated populations were quantified. Around 1000 cells were subjected to scRNA-seq analysis. The flow cytometry data are representative of at least three independent experiments, and error bars represent means ± SEM (n=3 for each group). **p < 0.01, ****p < 0.0001, Student’s *t* test.

To further systematically define the nature of exhausted monocytes generated *in vitro*, we performed scRNAseq analyses of monocytes cultured for 5 days with PBS or high dose LPS. UMAP analyses indicated that cultured control monocytes largely clustered into a homogenous population, represented with negative expression of Ly6C (**Figures 2A–C**), consistent with previous independent flow cytometry characterization of resting murine Ly6C^Neg^ monocytes reported by us and others (32, 33). In contrast, monocytes cultured for 5 days with high dose LPS were polarized into a majority Ly6C^++^ and a minority Ly6C^+^ population (**Figures 2A–C**). We independently confirmed a near complete shift of monocytes into the exhausted Ly6C^++^ and Ly6C^+^ populations and the disappearance of the resting Ly6C^Neg^ monocytes *via* flow cytometry analyses (**Figures 2D–G**). We also observed a decrease in the MFI of CD86, MHCII as well as an increase in the MFI of PD-L1 and CD38 in both Ly6C^+^ and Ly6C^++^ populations (**Supplementary Figures S2A–H**). Our data is consistent with emerging studies in both experimental animals and humans with sepsis and/or polymicrobial infections including COVID-19 patients, demonstrating the reduction of human non-classical monocyte population (murine Ly6C^Neg^ monocyte equivalent) and an increase of the exhausted monocyte population (human CD14^hi^, murine Ly6C^hi^ equivalent) (7, 14, 16).

Upon additional examination of selectively induced genes within LPS-exhausted monocytes, we identified additional representative genes independently reported to be highly elevated in septic monocytes from experimental animals and human septic patients, including *Cd38*, *Plac8, S100a8, Ptpn1, Lcn2, Ms4a3, Il1r2*, *Plaur*, and *Alox5* (34–39). Exhausted monocytes collected from septic patients not only expressed pathogenic inflammatory genes listed above, but also exhibit reduced expression of immune-enhancing genes such as CD86 (30). We identified the reduction of multiple genes involved in homeostasis and/or immune-enhancing processes including *Cd86, Cd200r, Apoe, Abca1, Trem2, Pik3r1, Smurf2*, and *Park2* (**Figure 2B**). Our data further validated our *in vitro* system in generating exhausted monocytes relevant to sepsis pathogenesis *via* repetitive high dose LPS challenges.

**Figure 3:**
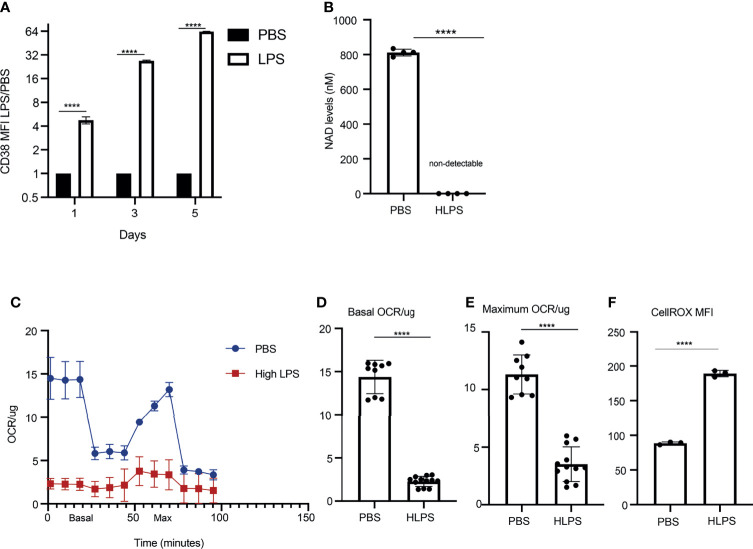
Exhausted monocytes express elevated levels of CD38 and exhibit compromised mitochondria respiration. BMDMs were stimulated with PBS or high dose LPS (100 ng/mL) for 5 days. **(A)** CD38 MFIs on Day 1,3 and 5 were analyzed with flow cytometry. The relative fold changes in MFIs of CD38 comparing LPS-treated *vs* PBS-treated monocytes were determined and plotted in the graphs. n=3. **(B)** NAD^+^ levels in monocytes treated with either PBS or high dose LPS were measured on Day 5 using a fluorescence-based assay. n=4. **(C)** OCR/ug was determined by Seahorse assay. n=3. **(D, E)** The basal and maximum OCR/ug levels were quantified. n≥9. **(F)** Intracellular ROS levels (n=3) were determined using flow cytometry. The data are representative of at least three independent experiments, and error bars represent means ± SEM. ****p < 0.0001, Student’s *t* test.

Persistent CD38 expression on monocytes has been shown to be involved in the establishment of sustained pathogenic inflammation, through depleting cellular source of NAD^+^ and compromising mitochondria function (40–42). Elevated CD38 expression was also implicated in propagating inflammation *via* inter-cellular communication (43). Upregulation of CD38 in septic patients has been reported previously (34), however detailed understanding on the role and regulation of CD38 in sepsis and monocyte exhaustion is still missing. We therefore validated the expression of CD38 protein on the surface of exhausted monocytes *via* flow cytometry analyses. Indeed, as shown in **Figure 3**, the levels of CD38 on monocytes kept on increasing upon repetitive LPS challenges (**Figure 3A**, **Supplementary Figure S1D**). We further tested cellular levels of NAD^+^ and observed a drastic reduction in LPS-exhausted monocytes as compared to control monocytes (**Figure 3B**). This is also consistent with a previous finding about reduced NAD^+^ in liver tissues from a Cecal Ligation and Puncture (CLP)-induced sepsis model (44). Seahorse assays revealed a significant compromise of mitochondria respiration in LPS-exhausted monocytes as compared to control monocytes as shown in the graph (**Figure 3C**). The basal and maximum OCR/ug in PBS *vs* high dose LPS are also shown in **Figures 3D, E**. Consequently, we documented a drastic increase of cellular ROS levels *via* staining with flow cytometry (**Figure 3F**). Collectively, our data revealed a sustained elevation of CD38 on *in vitro* exhausted monocytes, correlated with dysfunctional mitochondria and increased ROS, reminiscent of septic conditions from septic patients.

### Exacerbated STAT1 and KLF4 Activation Contribute To Monocyte Exhaustion

**Figure 4:**
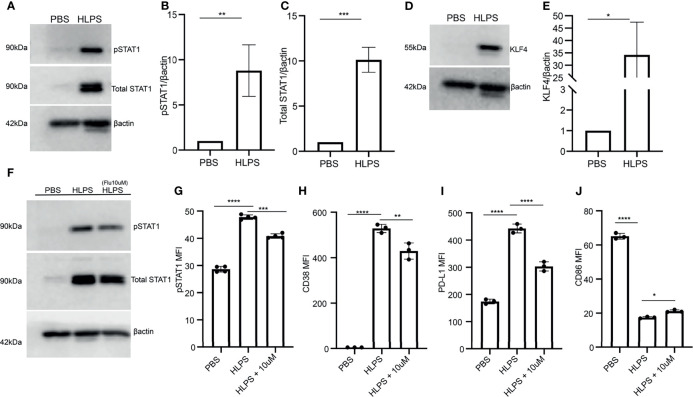
Exhausted monocytes exhibit sustained activation of STAT1. **(A–E)** BMDMs were stimulated with PBS or high dose LPS (100 ng/mL) for 5 days. The levels of phosphorylated STAT1 and total STAT1 were determined by Western blot, n=3. **(A)**. The relative levels of pSTAT1 **(B)** and STAT1 **(C)** were normalized to β-actin. The level of KLF-4 was determined by Western blot, n=3 **(D)**, and the relative level was normalized to β-actin **(E)**. **(F–J)** BMDMs were pre-treated with STAT1 inhibitor (Fludarabine 10 uM) on Day 0 for 2 hours, followed by stimulation with PBS or high dose LPS (100 ng/mL). The levels of pSTAT1 and STAT1 were determined using Western blot, n=3 **(F)** and flow cytometry, n=3 **(G)**. The expressions of CD38 **(H)**, PD-L1 **(I)** and CD86 **(J)** were analyzed with flow cytometry, n=3. The data are representative of at least three independent experiments, and error bars represent means ± SEM. *p < 0.05, **p < 0.01, ***p < 0.001, ****p < 0.0001, **(B, C, E)** Student’s *t* test and **(G–J)** one-way ANOVA.

Next, we explored the molecular mechanisms that may lead to monocyte exhaustion. Our work and others through computational analyses suggest that STAT1 may serve as a key node for driving the polarization of inflammatory classical monocytes in murine monocytes and human monocytes with polymicrobial infections including COVID-19 (14, 28). This is also consistent with additional experimental studies, reporting that STAT1 levels are upregulated in sepsis models (45); and that STAT1 deficient mice are protected from cecal-ligation and puncture-induced experimental sepsis (46). However, these previous phenotypic observations did not address the role of STAT1 in monocyte exhaustion. We then performed Western blot analyses comparing control and LPS-exhausted monocytes, and indeed confirmed a drastic elevation of both the phosphorylated STAT1 as well as total levels of STAT1 in LPS-exhausted monocytes as compared to control monocytes (**Figures 4A–C**).

In addition to STAT1, Kruppel-like factor 4 (KLF4) also plays a critical role in macrophage activation *via* LPS and IFNs (47). Role of KLF4 in inflammation is reported to be STAT-1 mediated (47, 48). Elevated KLF may not only work together with STAT1 in exacerbating pathogenic inflammation, but also serve as a suppressor of CD86 expression (47, 48). Hence, we investigated whether there is a coupled induction of KLF4 in exhausted monocytes. Indeed, we observe a sharp upregulation of KLF4 proteins in exhausted monocytes as compared to the control counterparts (**Figures 4D, E**).

To further test the causal role of STAT1 in driving monocyte exhaustion, we applied a selective STAT1 inhibitor to the monocyte culture. We pre-treated monocytes with 10uM fludarabine (STAT1 inhibitor) for 2 hours followed by 100ng/mL LPS treatment to attenuate STAT1 activation as shown in **Figures 4F, G**. We further observed that fludarabine pre-incubation partially decreased the expression of CD38 and PD-L1 induced by LPS, while also partially restored the expression of CD86 as compared to LPS exhausted monocytes (**Figures 4H–J**). Together, our data validated the role of STAT1 in driving monocyte exhaustion.

### Scavenging Cellular ROS Reduces STAT1 Activation and Monocyte Exhaustion

**Figure 5:**
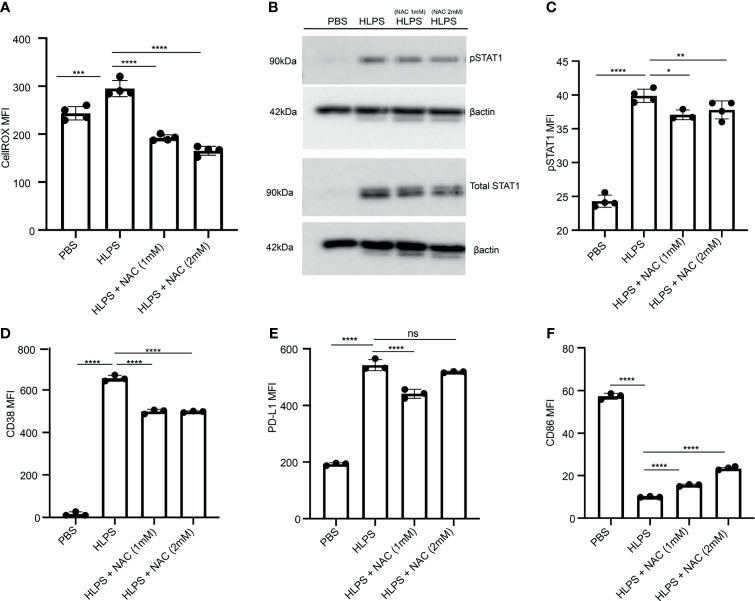
Scavenging ROS reduces STAT1 activation and monocyte exhaustion. BMDMs were stimulated with PBS or high dose LPS (100 ng/mL) for 5 days, and NAC was added to indicated cultures. **(A)** Intracellular ROS level was measured with flow cytometry, n=4. **(B, C)** The protein expression and phosphorylation of STAT1 was determined using Western blot, n=3 **(B)** and flow cytometry, n=4 **(C)**. **(D–F)** The MFIs of CD38 **(D)**, PD-L1 **(E)** and CD86 **(F)** were determined using flow cytometry, n=3. The data are representative of at least three independent experiments, and error bars represent means ± SEM. *p < 0.05, **p < 0.01, ***p < 0.001, ****p < 0.0001, ns: non-significant, one-way ANOVA.

Our data reveal that LPS-exhausted monocytes exhibit elevated CD38; compromised mitochondria function and elevated ROS. Previous reports showing that elevated ROS can further lead to STAT1 activation, thus potentially forming a sustained positive feedback loop (49, 50). Thus, we further tested whether scavenging cellular ROS may disrupt such positive feedback loop and reduce monocyte exhaustion. By applying the ROS scavenger N-acetyl-l-cysteine (NAC), we observed a significant drop in cellular ROS from LPS-exhausted monocytes (**Figure 5A**). Consequently, we observed that NAC application led to a reduction of LPS-mediated STAT1 activation (**Figures 5B, C**). NAC application also partially reduced CD38 and PD-L1 levels in LPS-exhausted monocytes (**Figures 5D, E**). On the other hand, NAC application partially restored the expression of CD86 in LPS-exhausted monocytes (**Figure 5F**).

### TRAM Is Required for the Induction of Monocyte Exhaustion

**Figure 6:**
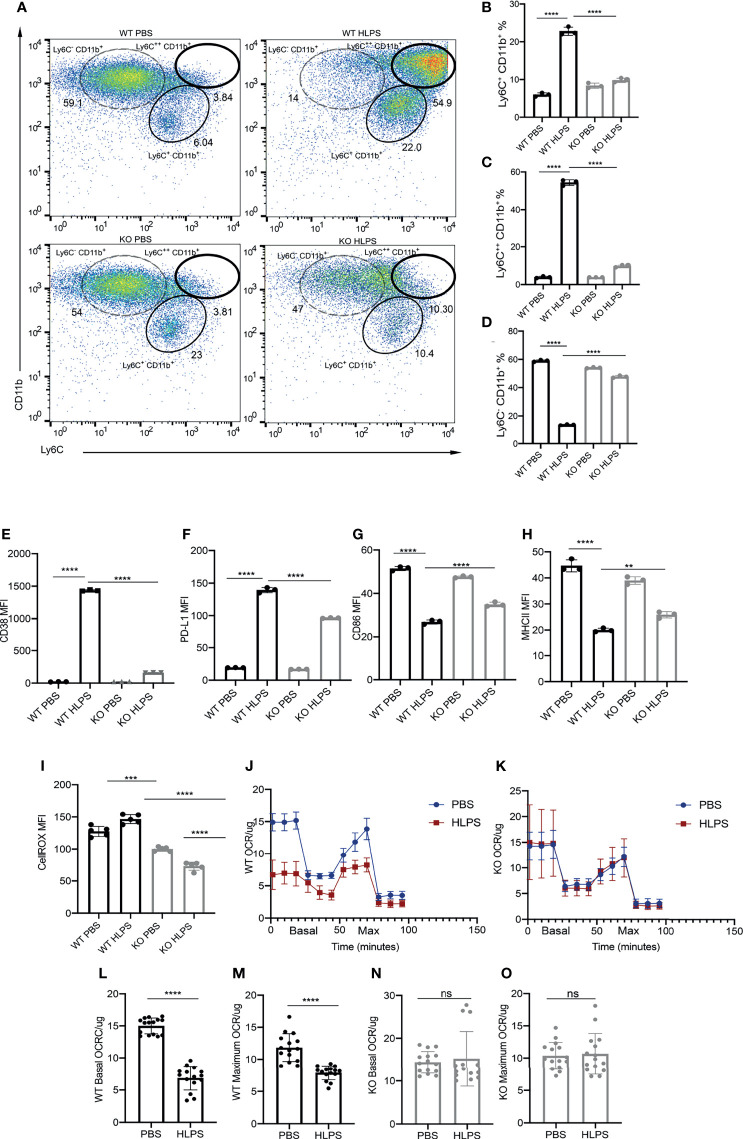
TRAM is required for the generation exhausted monocytes. BMDMs from WT and TRAM KO mice were treated with PBS or high dose LPS (100 ng/mL) for 5 days. **(A)** Ly6C^+^ CD11b^+^, Ly6C^++^ CD11b^+^ and Ly6C^-^ CD11b^+^ populations in the cell cultures were analyzed with flow cytometry. **(B–D)** The frequencies of indicated populations were quantified, n=3. **(E–H)** The MFIs of CD38 **(E)**, PD-L1 **(F)**, CD86 **(G)** and MHC-II **(H)** in WT and TRAM KO monocytes were determined with flow cytometry. **(I)** Intracellular ROS levels in WT and TRAM KO monocytes were determined with flow cytometry, n=3. **(J, K)** Mitochondrial OCR/ug levels in WT **(J)** and TRAM KO **(K)** monocytes were detected with Seahorse assay, n=5. **(L–O)** The basal and maximum OCR/ug levels in WT and TRAM KO were compared (n≥14). The data are representative of at least three independent experiments, and error bars represent means ± SEM. **p < 0.01, ***p < 0.001, ****p < 0.0001, ns: non-significant, **(B–I)** one-way ANOVA and **(L–O)** Student’s *t* test.

Given our previous report that TLR4 adaptor TRAM is responsible for STAT1 activation in exhausted neutrophils (51), we then further examined whether TRAM is responsible for monocyte exhaustion. To test this, we compared the exhaustion phenotype of WT and TRAM deficient monocytes challenged with repetitive LPS challenges. We observed that WT monocytes are readily exhausted into a majority Ly6C^++^ population and a minority Ly6C^+^ population (**Figures 6A–C**). In sharp contrast, TRAM deficient monocyte are resistant to LPS exhaustion, and largely remain as the Ly6C^-^ non-classical homeostatic population (**Figures 6A–D**). More strikingly, the induction magnitudes of CD38 and PD-L1 by LPS on TRAM deficient monocytes were drastically attenuated as compared to WT monocytes challenged by LPS (**Figures 6E, F**). On the other hand, the reduction of CD86 and MHCII by LPS on TRAM deficient monocytes was also dampened as compared to WT monocytes exhausted by LPS (**Figures 6G, H**).

We further measured the ROS levels and mitochondrial oxygen consumption rate (OCR/ug) in TRAM deficient monocytes. We observed that LPS challenge failed to induce cellular ROS levels in TRAM deficient monocytes (**Figure 6I**). Likewise, the OCR/ug were comparable in PBS *vs* LPS-treated TRAM deficient monocytes (**Figures 6J–O**). Collectively, our data reveal that TRAM is critically involved in the generation of exhausted monocytes by repetitive LPS challenges.

### TRAM Is Required for the Activation of STAT1 and KLF4 in Exhausted Monocytes

**Figure 7:**
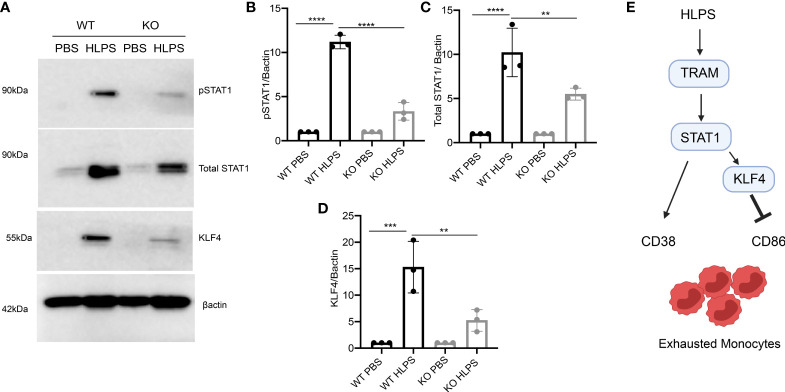
TRAM is required for the activation of STAT1/KLF4 involved in monocyte exhaustion. BMDMs from WT and TRAM KO mice were stimulated with PBS or high dose LPS (100 ng/mL) for 5 days. **(A)** The levels of pSTAT1, total STAT1 and KLF4 were determined by Western blot. **(B–D)** The relative levels of pSTAT1 **(B)**, STAT1 **(C)** and KLF4 **(D)** were normalized to β-actin. **(E)** Schematic illustration summarizing potential mechanism involved in monocyte exhaustion. The data are representative of at least three independent experiments, and error bars represent means ± SEM (n=3 for each group) **p < 0.01, ***p < 0.001, ****p < 0.0001, one-way ANOVA.

Since we observed a drastic upregulation of STAT1 and KLF4 in the exhausted monocytes, we further tested whether TRAM is required for their induction. We observed that the magnitudes of STAT1 and KLF4 activation were drastically attenuated in TRAM KO monocytes challenged with LPS, as compared to WT monocytes exhausted by LPS treatments (**Figures 7A–D**). Together, our data show that the monocyte exhaustion generated *via* repetitive LPS challenges was dependent upon TRAM-mediated activation of STAT1. Finally, a schematic illustration of the potential molecular mechanisms partaking in monocyte exhaustion is shown in **Figure 7E**.

## Discussions

In this report, we collected data that support the generation of exhausted Ly6C^hi^ monocytes exhibiting gene expression profiles of pathogenic inflammation and immuno-suppression characteristic of septic monocytes *via* repetitive challenges with high dose LPS. We identified CD38 as a novel marker for exhausted monocytes associated with depleted cellular NAD^+^, compromised mitochondria function and elevated cellular ROS. Our mechanistic study further revealed that TRAM-mediated exacerbation of STAT1 activation is responsible for sustained CD38 expression and monocyte exhaustion.

Our findings expand and complement previous experimental systems in examining the fundamental dynamics of innate immune training and memory. Although earlier works predominately focused on endotoxin tolerance as reflected in reduced cytokine expressions from monocytes repetitively challenged with LPS (3, 16), some limited studies reported that “tolerant” cells can still robustly respond to LPS and express “non-tolerizable” genes (20, 52, 53). Earlier time-course studies with monocytes reported that upon LPS challenge, human monocytes initially express CD86 at an earlier time point and followed by a reduction of CD86 expression at later time points (54, 55). Our current study provided a systems analyses of monocytes following repetitive and prolonged LPS challenges. We confirmed previously findings that although LPS challenge initially induced the expression of CD86, repetitive and prolonged LPS challenges led to a reduction of CD86, consistent with the “tolerant” phenotype. Our previous study employing the same culture system also reported the development of tolerance in terms of suppressed expression of immune-enhancing inflammatory genes such as IL-12 and CCR5 when monocytes were persistently challenged with high dose LPS (56). It was well-recognized that not all genes are suppressed in “tolerant” monocytes, and many pathogenic inflammatory genes can still be robustly induced under “tolerant” settings (20, 56). Thus, the terminology of “tolerance” coined several decades of ago cannot fully capture the dichotomy of pathogenic inflammation and immuno-suppression phenotype of septic monocytes with persistent challenges of endotoxin, which may be better captured by the terminology of “exhaustion”. Indeed, monocytes with repetitive LPS challenges still maintain robust induction of PD-L1. Our scRNAseq analyses also revealed the induction of SLPI, an anti-inflammatory molecule; IL-1R2, an antagonist of IL-1 signaling. Both of SLPI and IL-1R2 were shown to be elevated in septic monocytes collected from human patients (57, 58). Collectively, the elevation of immuno-suppressors such as PD-L1, SLPI and IL-1R2 and reduction of co-stimulatory molecules such as CD86 contribute to the immuno-suppressive phenotype characteristics of exhausted monocytes observed in clinical sepsis patients (11, 31, 57, 58).

In addition to well-defined immuno-suppressive molecules mentioned above, the exhausted monocytes generated *in vitro* also expressed representative pathogenic inflammatory genes identified in human septic patients including *S100a8*, *Lcn2*, and *Alox5* (35, 38). Furthermore, we identified elevated expression of *Plac8*, *Plaur* and *Ms4a3* in the exhausted monocytes, consistent with recent single cell sequencing analyses and machine learning characterization studies of monocytes from human septic patients (14, 36–38). The inductions of pathogenic inflammatory genes are consistent with both murine and human studies. On the other hand, the independent scRNAseq analyses of human septic monocytes also revealed a subset of monocytes with reduced expression of class II MHC (37), consistent with the immuno-suppressive phenotype representative of monocyte exhaustion. Despite these important phenotypic findings, future detailed comparative studies are needed to better define evolutionarily conserved monocyte exhaustion phenotypes from mice to humans subjected to sepsis. Further, independent validation at protein levels should be performed to confirm the targets obtained from the scRNAseq-based gene expression studies. Together, our systems analyses reveal the generation of exhausted monocytes with cardinal features of pathogenic inflammation coupled with immuno-suppression representative of monocytes from septic patients.

Complementing our gene expression analyses, our flow cytometry-based measurements of key cell-surface protein molecules further validated key features of pathogenic inflammation and immuno-suppression. Upon exhaustion, the homeostatic non-classical Ly6C^-^ monocyte population almost completely disappeared, consistent with clinical studies of septic patients reporting the reduction of human counter-part of CD14^lo^CD16^hi^ non-classical monocytes (37). Intriguingly, COVID-19 patients may experience similar monocyte exhaustion with a drastic reduction of non-classical monocyte population (14, 15), suggesting monocyte exhaustion as a universal mechanism underlying the pathogenesis of polymicrobial sepsis. It is also worth noting that there are two subsets of exhausted monocytes (the classical Ly6C^++^ and the intermediate Ly6C^+^), although both share similar features of pathogenic inflammation/immuno-suppression markers. Our findings are consistent with recent human patients-based studies revealing the expansion of two subsets of human monocytes (classical and intermediate) in sepsis as well as severe COVID-19 patients (14, 15). The differential contributions of these two exhausted monocyte subsets to the severity of sepsis, however, will require future studies with animal models.

Among cell surface pathogenic inflammatory molecules, we identified a robust and persistent induction of CD38, an important enzyme in depleting both intra-cellular and inter-cellular NAD^+^ (21, 23). Although moderate and transient expression of CD38 may be beneficial, exacerbated and prolonged CD38 expression was implicated in depleting cellular NAD^+^ and causing run-away pathogenic inflammation (42). The previous study regarding CD38 regulation used the single LPS challenge system, and showed a transient induction of CD38 (42). In sharp contrast, under the prolonged septic stimulatory condition, we observed a sustained induction of CD38. Such sustained CD38 expression may be responsible for the depletion of cellular NAD^+^, leading to the exhaustion phenotype we observed in this current study. Indeed, we observed a drastic reduction of mitochondria respiration and elevated cellular ROS in monocytes with prolonged LPS challenges, another key feature of exhausted monocytes observed independently from septic models (40, 41, 59). Mitochondrial dysfunction has been directly associated with organ dysfunction in animal sepsis as well as septic patients (40). Excessive oxidative stress and inflammatory mediators inhibit mitochondrial respiration, destruct mitochondrial protein and lipid membrane and hence, are key pathogenesis for mitochondrial dysfunction in sepsis (40, 60). In age-related diseases, CD38 was shown as an important NAD-degrading enzyme further leading to mitochondrial dysfunction and CD38 deficiency maintains NAD^+^/NADH ratio in mitochondria (23). Our data revealed that CD38 may serve as a novel cell-surface indicator of exhausted monocytes that may sustain and propagate pathogenic inflammation associated with sepsis.

Our current study not only systematically revealed the pathogenic inflammatory and immuno-suppressive features of exhausted monocytes, but also identified potential underlying mechanisms that involve sustained exacerbation of STAT1 activation. Our finding is consistent with a previous phenotypic report that demonstrated higher survival rates in septic mice with STAT1 deficiency as compared to WT controls (80% *vs* 10%) (46). Another independent study suggests that STAT1 may synergize with KLF4 in further skewing pathogenic inflammation (47). STAT1 was shown to be important in the upregulation of PD-L1 (61, 62) while KLF4 was shown to suppress CD86 expressions (63). Consistent with these earlier reports, we observed robust elevation of both STAT1 and KLF4 in exhausted monocytes. KLF4 may perform both pro- as well as anti-inflammatory functions, likely modulated by its context-dependent binding partners. It remained to be tested whether STAT1-coupled KLF4 may over-ride the beneficial effects of stand-alone anti-inflammatory effects of KLF4. The detailed co-operation among STAT1 and KLF4 in the context of monocyte exhaustion requires extensive future studies. Our current approach of applying STAT1 inhibitor partially attenuated monocyte exhaustion. We realize the limitation of the inhibitor approach due to its potential off-target effects and such approach may not be sufficient in finely characterize the involvement of STAT1 as well as intertwined ROS generation during the complex development of various monocyte exhaustion phenotypes. To better define complex mechanisms involved in the dichotic modulation of genes involved in pathogenic inflammation as well as immuno-suppression, future independent approaches with genetic STAT1 deletion would be warranted to further precisely determine the contribution of STAT1 during the generation of exhausted monocytes.

Our study also revealed the important contribution of TRAM adaptor molecule in the generation of exhausted monocytes. TRAM is a less studied TLR4 adaptor molecule (64), that may coordinate TLR4 signaling dynamics, in competition and/or cooperation with other known adaptor molecules such as MyD88 and TRIF. Both MyD88 and TRIF have been shown to facilitate the generation of immune-enhancing monocytes (65–67). Innate stimulants such as beta-glucan can generate immune-enhancing trained immunity through MyD88 and/or TRIF dependent activation of PI3K pathway, and exert protective effects in experimental sepsis (68, 69). Some limited mechanistic studies suggest that these adaptor molecules may competitively function downstream of TLR4 and finely modulate monocyte activation dynamics ranging from priming, tolerance to exhaustion (56, 70–72). While TRAM may coordinate with TRIF in monocytes under low-grade inflammatory conditions, and facilitate the priming/immune-enhancing effects (56), our current data suggest that TRAM may also be uniquely involved in facilitating the generation of exhausted monocytes, perhaps independent of TRIF, which requires further clarification with detailed studies. Our data suggest a distinct role of TRAM in selectively propagating monocyte exhaustion *via* facilitating the sustained activation of STAT1 and KLF4. We demonstrated that TRAM deficiency drastically attenuated the induction of both STAT1 and KLF4 by LPS, and kept monocytes predominantly within the Ly6C^-^ non-classical resting state. TRAM deficient monocyte also maintained proper mitochondria respiration following repetitive LPS challenges and balanced expression of CD86/PD-L1. Our findings not only reveal a unique mechanism for monocyte exhaustion, but also suggest that TRAM may serve as a promising target for mitigating monocyte exhaustion and preventing sepsis pathogenesis.

## Data Availability Statement

scRNAseq data sets were deposited in the Genebank with the accession number GSE182355. All other relevant data are presented in this article.

## Ethics Statement

The animal study was reviewed and approved by Virginia Tech Institutional Animal Care and Usage Committee (IACUC).

## Author Contributions

KP and LL designed experiments. KP designed the experiments, performed the studies, analyzed the data and wrote the manuscript. ZY performed the scRNAseq analyses. SG assisted with manuscript preparation. LL designed and supervised the studies, wrote the manuscript. All authors contributed to the article and approved the submitted version.

## Funding

This study is partially supported by the grant from National Institutes of Health R01AI136386.

## Conflict of Interest

The authors declare that the research was conducted in the absence of any commercial or financial relationships that could be construed as a potential conflict of interest.

## Publisher’s Note

All claims expressed in this article are solely those of the authors and do not necessarily represent those of their affiliated organizations, or those of the publisher, the editors and the reviewers. Any product that may be evaluated in this article, or claim that may be made by its manufacturer, is not guaranteed or endorsed by the publisher.

## Supplementary Material

Supplementary Figure 1 Representative histograms of monocyte tolerant and exhaustion markers. BMDMs from WT mice were treated with PBS or high dose LPS (100ng/mL) for 5 days. **(A–D)** Representative histograms illustrating shifts in surface MFIs of CD86 **(A)**, MHCII **(B)**, PD-L1 **(C)** and CD38 **(D)** in live cells obtained *via* flow cytometry were presented.

Supplementary Figure 2 Ly6C^+^ and Ly6C^++^ monocyte populations show exhausted phenotypes. BMDMs from WT mice were treated with PBS or high dose LPS (100ng/mL) for 5 days. **(A–D)** Surface expressions of CD86 **(A)**, MHCII **(B)**, PD-L1 **(C)** and CD38 **(D)** within the Ly6C^+^ CD11b^+^ population were determined with flow cytometry. **(E–H)** Similarly, expressions of CD86 **(E)**, MHCII **(F)**, PD-L1 **(G)** and CD38 **(H)** within the Ly6C^++^ CD11b^+^ population were determined with flow cytometry. The data are representative of at least three independent experiments, and error bars represent means ± SEM (n=3 for each group). ***p < 0.0001, Student’s *t* test.
