# Supplementary material for: Development of Exhausted Memory Monocytes and Underlying Mechanisms
Source: Front Immunol. 2021 Oct 28;12:778830. doi: 10.3389/fimmu.2021.778830 (PMC8583871; doi:10.3389/fimmu.2021.778830)
Supplement: Supplementary Figure 1 — Representative histograms of monocyte tolerant and exhaustion markers. BMDMs from WT mice were treated with PBS or high dose LPS (100ng/mL) for 5 days. (A–D) Representative histograms illustrating shifts in surface MFIs of CD86 (A), MHCII (B), PD-L1 (C) and CD38 (D) in live cells obtained via flow cytometry were presented. [file Presentation_1.pdf]

Supplemental Materials

Development of exhausted memory monocytes and underlying mechanisms

Kisha Pradhan[1], Ziyue Yi[2], Shuo Geng[1], Liwu Li[1,2]

[1]Department of Biological Sciences, Virginia Tech, Blacksburg, VA24061; [2]Graduate Program of Genetics, Biotechnology and Computational Biology, Virginia Tech, Blacksburg, VA24061

Running Title: Generation of exhausted monocyte memory

Keywords: Monocyte memory, exhaustion, pathogenic inflammation, CD38, TRAM

*Correspondence:

Liwu Li, Ph.D

970 Washington Street, Virginia Tech, Blacksburg, VA 24061-0910 Email: lwli@vt.edu

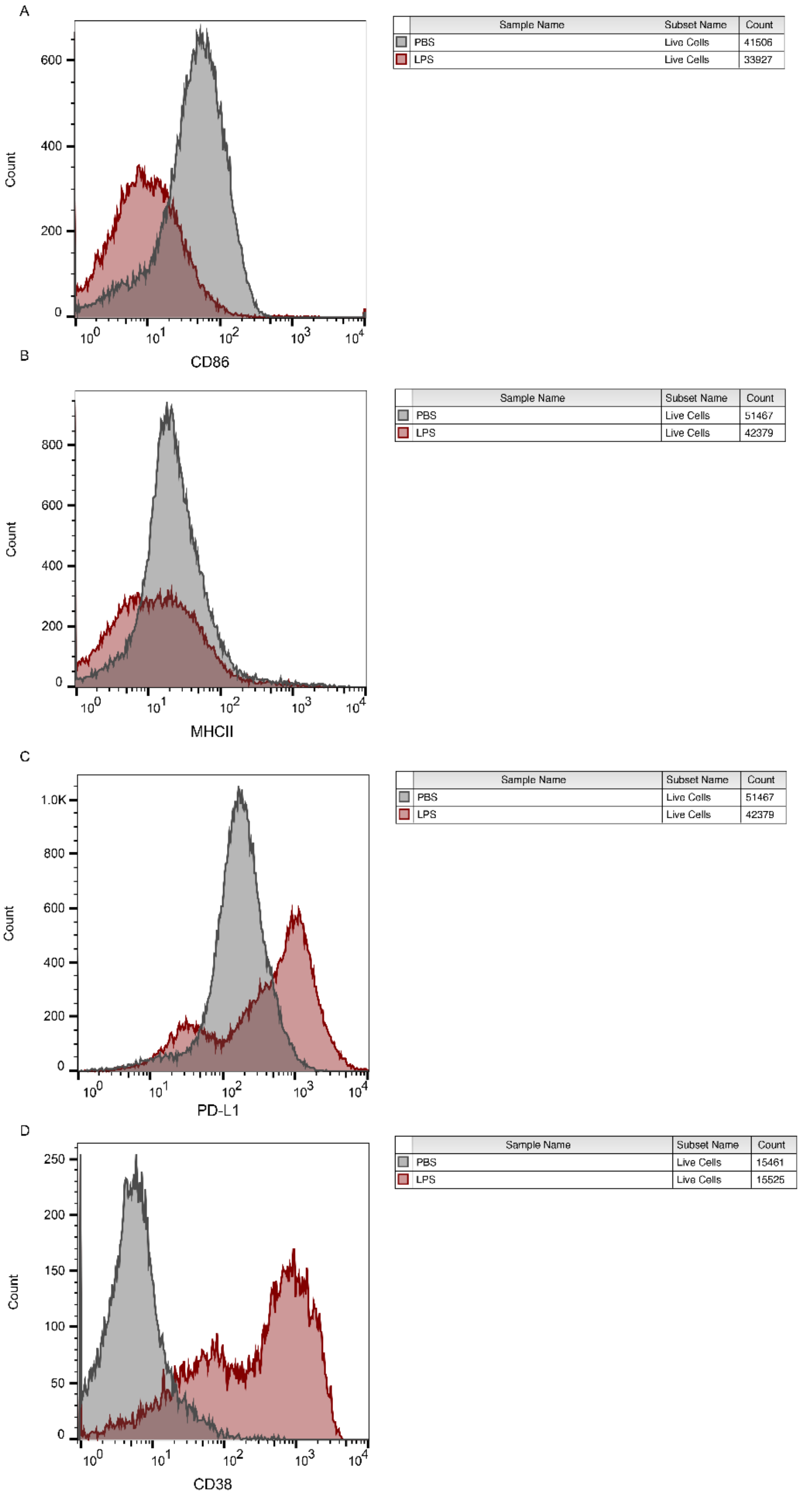

Supplementary Figure S1. Representative histograms of monocyte tolerant and exhaustion markers. BMDMs from WT mice were treated with PBS or high dose LPS (100ng/mL) for 5 days. (A-D) Representative histograms illustrating shifts in surface MFIs of CD86 (A), MHCII (B), PD-L1 (C) and CD38 (D) in live cells obtained via flow cytometry were presented.

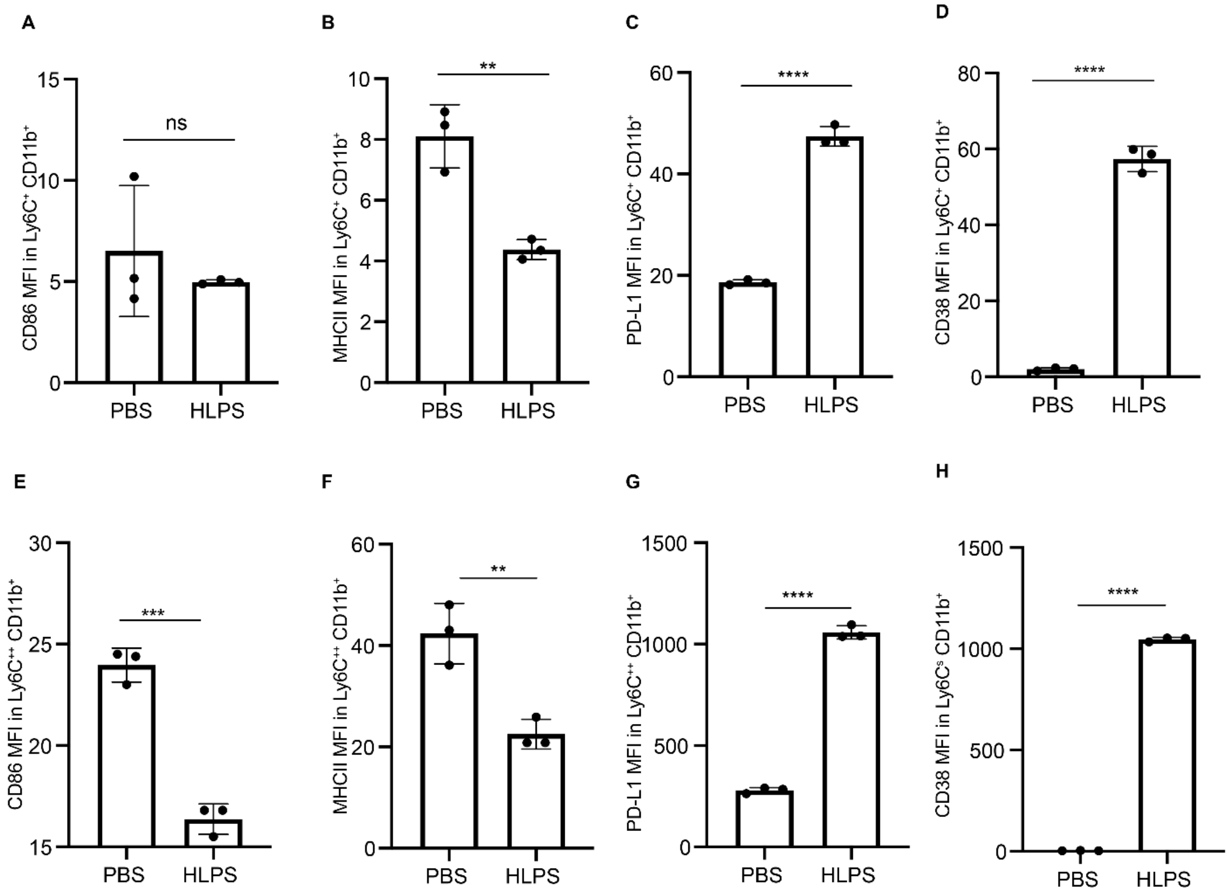

Supplementary Figure S2. Ly6C^{+} and Ly6C^{++} monocyte populations show exhausted phenotypes

BMDMs from WT mice were treated with PBS or high dose LPS (100ng/mL) for 5 days. (A-D) Surface expressions of CD86 (A), MHCII (B), PD-L1 (C) and CD38 (D) within the Ly6C^{+} CD11b^{+} population were determined with flow cytometry. (E-H) Similarly, expressions of CD86 (E), MHCII (F), PD-L1 (G) and CD38 (H) within the Ly6C^{++} CD11b^{+} population were determined with flow cytometry. The data are representative of at least three independent experiments, and error bars represent means ± SEM (n=3 for each group). ****p < 0.0001, Student's *t* test.
